# Evaluation of Physical Fitness in Water Polo Players According to Playing Level and Positional Role

**DOI:** 10.3390/sports6040157

**Published:** 2018-11-28

**Authors:** Petros G. Botonis, Argyris G. Toubekis, Theodoros I. Platanou

**Affiliations:** Division of Aquatic Sports, School of Physical Education and Sports Science, National and Kapodistrian University of Athens, 17237 Athens, Greece; atoubekis@phed.uoa.gr (A.G.T.); tplatan@phed.uoa.gr (T.I.P.)

**Keywords:** muscle strength, endurance, fitness profile, team-sports performance

## Abstract

Background: We aimed to investigate whether water polo players of different playing levels and positions differ in fitness parameters (i.e., strength, aerobic endurance, and anaerobic potential). Methods: Twenty-four water polo players were assigned to international- (IL) and national-level (NL) groups or to centers and peripherals. At the beginning of preseason training, maximal bench press strength was measured and a speed–lactate test (5 × 200m) was performed to determine the speed corresponding to lactate concentrations of 4.0 (V4), 5.0 (V5), and 10.0 (V10) mmol·L^−1^. Results: Maximal muscular strength was similar between international- and national-level water polo players, but it was higher in centers than in peripherals (109.2 ± 12.2 kg vs. 96.9 ± 8.5 kg, *p* = 0.007). IL players showed higher V4, V5, and V10 compared to NL players (V4, IL: 1.27 ± 0.04 m·s^−1^ vs. NL: 1.17 ± 0.06 m·s^−1^), (V5, IL: 1.33 ± 0.03 m·s^−1^ vs. NL: 1.22 ± 0.05 m·s^−1^), and V10 (IL: 1.50 ± 0.31 vs. NL: 1.35 ± 0.06 m·s^−1^) (*p* < 0.01)). However, no significant differences were detected between centers and peripherals inV4, V5, and V10. Conclusions: We suggest that V4, V5, and V10 distinguish playing level in water polo, whereas they are comparable between playing positions. Although maximal strength is similar between playing levels, it is different between playing positions.

## 1. Introduction

Water polo players participate in several high-intensity bouts separated by lower-intensity efforts [1,2], suggesting that high levels of strength and aerobic and anaerobic capacity are essential for successful participation in elite water polo leagues [3]. Recent evidence suggests that the swimming velocity corresponding to 4.0 mmol·L^−1^ (V4), 5.0 mmol·L^−1^ (V5), and 10.0 mmol·L^−1^ (V10) as well as the differential velocity between 10–5 mmol·L^−1^ (V10–V5) are indicators of the aerobic and anaerobic potential of water polo players [4,5,6]. Therefore, the abovementioned indicators are sensitive indices for in-game water polo performance [6]. Although it is reasonable to expect that the abovementioned indicators likely discriminate among players of national and international levels, no study has compared the strength level as well as the physiological characteristics depicting both aerobic and anaerobic power of water polo players of different playing standards.

Moreover, the demands of water polo are role dependent and mainly linked to the required individual skills [7]. A number of early and more recent motion analysis studies indicate that center defenders and center forwards may execute different movement patterns compared to peripherals. The former (centers) have been found to engage in more dynamic body contacts/wrestling, whereas the latter (peripherals) have been shown to perform a greater number of swimming bouts [7,8]. As such, coaches design training plans where players perform different exercise tasks according to their playing position, aiming at improving individuals’ specific game requirements. To date, however, it is unknown whether the observed differences in movement patterns among players of different positional roles mirror possible differences in muscular strength as well as in aerobic and anaerobic potential.

Based on the abovementioned analysis, the recording of both muscular strength level and fitness indices of elite male water polo players appears to be of great scientific and practical interest for two main reasons. First, it provides a scientific picture of the upper limits of the adaptations derived from long-term water polo training. Second, because it provides scientific evidence to coaches regarding the strength level and fitness profile of modern water polo players, thus offering evidence regarding water polo players’ selection and profiling.

Therefore, the purpose of the present study was to examine whether international-level (IL) and national-level (NL) players as well as players of different positional roles (centers and peripherals) differ in muscular strength and in important indicators of aerobic and anaerobic potential, such as V4, V5, V10, and V10–V5. It was hypothesized that IL players would present higher strength and aerobic and anaerobic potential compared to the NL players. It was further hypothesized that centers and peripherals would demonstrate similar fitness levels due to the similar overall physiological load applied on the players during competition. Nonetheless, it was expected that centers would exhibit greater muscular strength compared to peripherals.

## 2. Materials and Methods

### 2.1. Participants

Twenty-four male water polo players participating in three different clubs in the top-level division of the Greek Championship took part in the study and were assigned to international-level (n = 12, age: 27.7 ± 3.9 years, stature: 189.2 ± 4.2 cm, body mass: 91.6 ± 8.5 kg) and national-level (n = 12, age: 27.4 ± 5.5 years, stature: 184.5 ± 7.1 cm, body mass: 87.5 ± 10.8 kg) players. Accordingly, they were assigned to two groups of different playing positions (centers, n = 12, age: 29.5 ± 4.7 years, stature: 190.6 ± 4.1 cm, body mass: 96.9 ± 7.7 kg; peripherals, n = 12, age: 26.3 ± 4.3 years, stature: 183.4 ± 6.1 cm, body mass: 82.7 ± 6.4 kg). All players were at the top level and trained on a daily basis. From the selected IL players, 10 of them were regular members of the Greek National Team which competed in Olympic Games of London and Rio (2012, 2016), whereas the other two were members of the Spanish and Croatian National Teams competing in the same Olympic Games. NL players were elite players participating in the Greek top-level division with more than five years of training and playing experience. Informed consent was obtained from all players before testing. The experimental protocol was approved by the faculty review board of the University of Athens and conformed to the Declaration of Helsinki.

### 2.2. Testing Procedures

At the beginning of preseason training, players’ fitness level was evaluated through an incremental swimming test (5 × 200 m) [9]. After a 10-min standardized warm-up, participants swam five repetitions of 200 m, in an outdoor 25-m pool, at intensities corresponding to 60%, 70%, 80%, 90%, and 100% of maximum speed, with a 5-min passive recovery between each effort. Fingertip blood samples were taken after each 200-m repetition and were immediately analyzed using the reflectance photometry-enzymatic reaction method (Accusport, Boehringer, Germany). The speeds corresponding to lactate concentrations of 4.0, 5.0, and 10.0 mmol·L^−1^ (V4, V5, and V10) were calculated from the speed–lactate curve by interpolation of a second-order polynomial function. The lactate tolerance ability was defined as the differential velocity between blood lactate concentrations of 5.0 and 10.0 mmol·L^−1^ (V10–V5) [10]. On a separate day, a one repetition maximum (1RM) on bench press was measured for the evaluation of maximal strength. A 2-min recovery between each effort and 2.5-kg increments, respectively, were used. A specific 10-min warm-up procedure using light weights and allowing participants to perform 10–15 repetitions and stretching exercises was followed before the initiation of 1 RM measurement. The players were familiar with bench press exercises as part of their regular strength training program in the previous season.

### 2.3. Statistical Analysis

Before using parametric statistical test procedures, the normality of the data was verified by the Shapiro–Wilk test. Analysis of variance for repeated measures on independent samples (IL vs. NL players and centers vs. peripherals) was used to detect differences in strength level, V4, V5, V10, and V10–V5. Tukey’s test was employed to allocate post-hoc specific differences. A *t*-test was also employed to detect differences between groups in anthropometric characteristics. As a measure of effect size, Cohen’s d was computed by calculating the mean difference for the groups and then dividing the result by the pooled standard deviation. The magnitude of each effect size was classified as trivial (*d* < 0.2), small (*d* = 0.2–0.6), moderate (*d* = 0.6–1.2), large (*d* = 1.2–2.0), very large (*d* = 2.0–4.0), and extremely large (*d* > 4.0) [11]. Data are expressed as mean ± standard deviation (SD). Statistica v.8.0 statistical package (Statsoft, Tulsa, OK, USA) was used for the analyses and the significance level was set at *p* < 0.05.

## 3. Results

**Figure 1:**
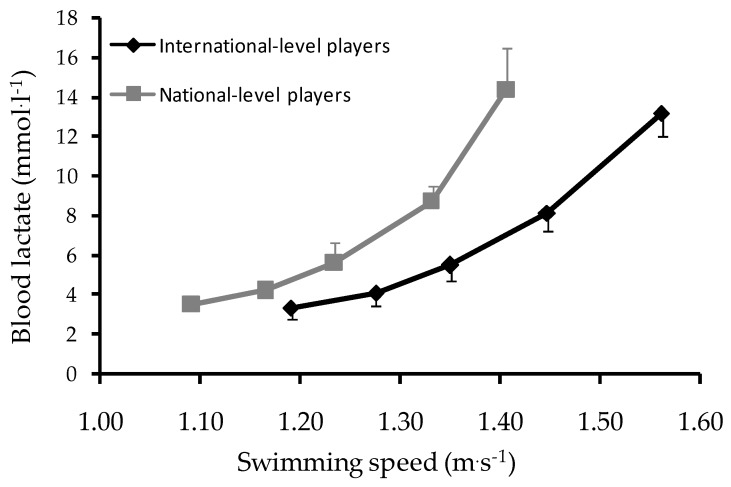
Speed–lactate curve for international- (n = 12) and national-level (n = 12) water polo players (mean ± SD). Note that a significant difference between international-level and national-level water polo players in V4, V5, and V10 was observed (*p* < 0.01).

IL water polo players had similar age (*p* = 0.87) and body mass (*p* = 0.29), but they were taller compared to NL players (*p* = 0.05, *d* = 0.7). Maximal muscular strength was similar between IL and NL water polo players (103.5 ± 11.6 kg vs. 102.7 ± 13.0 kg, *p* = 0.87). The speed–lactate curve of IL and NL players is presented in Figure 1. IL players showed higher V4 (1.27 ± 0.04 m·s^−1^ vs. 1.17 ± 0.06 m·s^−1^, *p* < 0.01, *d* = 1.75), V5 (1.33 ± 0.03 m·s^−1^ vs. 1.22 ± 0.05 m·s^−1^, *p* < 0.01, *d* = 2.50), and V10 (1.50 ± 0.31 m·s^−1^ vs. 1.35 ± 0.06 m·s^−1^, *p* < 0.01, *d* = 3.27) than NL players. Nonetheless, V10–V5 was similar between groups of different playing levels (IL: 0.16 ± 0.03 m·s^−1^ vs. NL: 0.13 ± 0.05 m·s^−1^, *p* > 0.05).

**Figure 2:**
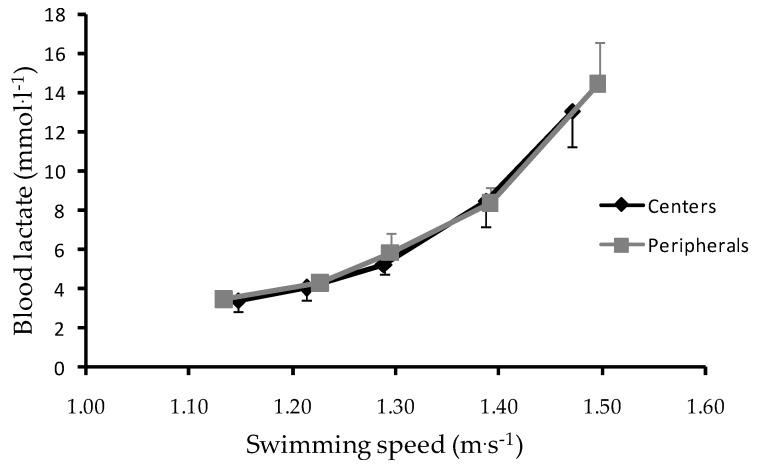
Speed–lactate curve for center (n = 12) and peripheral (n = 12) water polo players (mean ± SD).

Regarding playing position, centers were heavier (*p* = 0.00 and *d* = 2.0) and taller (*p* = 0.00 and *d* = 1.4) than peripherals, while both groups had a similar age (*p* = 0.09, *d* = 0.7). Maximal muscular strength was greater in centers than in peripherals (109.2 ± 12.2 kg vs. 96.9 ± 8.5 kg, *p* = 0.007, *d* = 1.2). The mean speed–lactate curve for centers and peripherals is depicted in Figure 2. Analysis of variance showed no difference between centers and peripherals in any of the measured parameters (V4 1.23 ± 0.09 m·s^−1^ vs. 1.22 ± 0.06 m·s^−1^, V5 1.28 m·s^−1^ ± 0.08 vs. 1.28 ± 0.06 m·s^−1^, V10 1.42 ± 0.10 m·s^−1^ vs. 1.42 ± 0.08 m·s^−1^, *p* > 0.05).

## 4. Discussion

The present study aimed at examining whether water polo players of different playing standards and positional roles differ in strength and aerobic and anaerobic potential. The principal findings of the study are: (a) muscular strength is similar between IL and NL water polo players, but IL players demonstrate higher aerobic (V4) and anaerobic (V5 and V10) potential compared to NL players, and (b) centers demonstrate a higher strength level but similar aerobic and anaerobic potential compared to peripherals.

Due to the high presence of dynamic actions that occur in water polo match-play (body contacts/wrestling, fast swimming, offence or defence actions), an adequate strength level is essential for water polo players [3]. Accordingly, dry-land strength training has been extensively recommended in the literature, as it induces considerable gains in the maximal strength level of elite or top-class water polo players [4,12,13]. Previous studies in land-based team-sports have found higher values of absolute maximal strength in international-level athletes compared to national-level athletes [14,15]. However, the present study shows that absolute values of maximal strength do not discriminate among international- and national-level water polo players. Most likely, the present findings indicate that other parameters of strength not presently measured such as the specific in-water strength of upper and/or lower limbs are more sensitive indicators of water polo performance.

Moreover, the total duration of the game along with the high frequency of high-intensity bouts separated by efforts of short duration imply that both aerobic and anaerobic potential are crucial for in-game water polo performance. According to our first hypothesis, we expected that V4, V5, V10, as well as the index of lactate tolerance (V10–V5) would be different between IL and NL players and our findings partially confirmed our assertions. Indeed, we observed that the aerobic and anaerobic potential of IL players was higher by 8–10% compared to the NL players. Interestingly, the V4 values presently observed are similar to those observed in a previous study (1.29–1.35 m·s^−1^) [4] regarding top-class water polo players and are also comparable to those reported by Fernandes et al. [16] concerning long-distance swimmers. Nevertheless, no difference was found for lactate tolerance. The absence of differences in lactate tolerance between IL and NL players implies that top-class players do not present significant lactate tolerance training adaptations due to the fact that activities during water polo matches do not require high levels of lactate tolerance from the participants [17], and as such, this physical ability is probably of lower priority during training.

We also found that centers are heavier and taller compared to peripherals. This finding is in accordance with previous observations regarding the anthropometrical differences between playing positions [17] and indicate that anthropometrics can discriminate among positional requirements in water polo. Additionally, in contrast to empirical practice, where often centers and peripherals execute different exercise tasks in order to improve their physical abilities, we found that centers and peripherals demonstrated similar aerobic and anaerobic potential, but centers presented higher levels of strength. Indeed, motion analysis studies of the game suggest that peripherals execute more swimming sprints compared to centers throughout competitive games, whereas centers complete more body contacts and/or wrestling than peripherals [7,8]. As such, a greater level of upper-body strength is required from centers to cope with the physical demands of body contacts and wrestling. Most likely, the absence of differences in selected fitness parameters between centers and peripherals depicts that, despite the differences in movement patterns, the overall physiological load imposed on players is similar, regardless of position.

The present study has practical importance because it demonstrates that (a) absolute strength level, as measured by the bench press, is not a distinguishing factor between playing levels in men’s water polo, (b) aerobic and anaerobic potential is considerably different between playing level, and (c) centers show higher absolute strength but similar V4, V5, and V10 compared to peripherals. These results provide information regarding the fitness requirements of top-class water polo players and can contribute to talent selection and identification.

## 5. Conclusions

In summary, our study suggests considerable differences in aerobic and anaerobic potential between IL and NL water polo players, whereas the respective fitness indices are comparable for centers and peripherals. More research is needed to identify to role of different strength measures in discriminating water polo players of different playing level.

## References

[1] Platanou T. (2004). Time motion analysis of the international level water polo players. J. Hum. Mov. Stud..

[2] Platanou T., Geladas N. (2006). The influence of game duration and playing position on intensity of exercise during match-play in elite water-polo players. J. Sports Sci..

[3] Botonis P.G., Toubekis A.G., Platanou T.I. (2018). Physiological and tactical on-court demands of water polo. J. Strength Cond. Res..

[4] Botonis P.G., Toubekis A.G., Platanou T.I. (2016). Concurrent strength and interval endurance training in elite water polo players. J. Strength Cond. Res..

[5] Botonis P.G., Toubekis A.G., Platanou T.I. (2016). Physical performance during water-polo matches: The effect of the players’ competitive level. J. Hum. Kinet..

[6] Botonis P.G., Toubekis A.G., Terzis G.D., Geladas N.D., Platanou T.I. (2016). Performance decrement and skill deterioration during a water polo game are linked with the conditioning level of the athletes. J. Strength Cond. Res..

[7] D’Auria S., Gabbett T. (2008). A time-motion analysis of international women’s water polo match-play. Int. J. Sports Physiol. Perform..

[8] Tan F., Polglaze T., Dawson B. (2009). Activity profiles and physical demands of elite women’s water polo match play. J. Sports Sci..

[9] Tsekouras Y.E., Kavouras S.A., Campagna A., Kotsis Y.P., Syntossi S.S., Papazoglou K., Sidossis L.S. (2005). The anthropometrical and physiological characteristics of elite water polo players. Eur. J. Appl. Physiol..

[10] Pyne D.B., Lee H., Swanwick K.M. (2001). Monitoring the lactate threshold in world-ranked swimmers. Med. Sci. Sports Exerc..

[11] Hopkins W.G., Marshall S.W., Batterham A.M., Hanin J. (2009). Progressive statistics for studies in sports medicine and exercise science. Med. Sci. Sports Exerc..

[12] Ramos Veliz R., Requena B., Suarez Arrones L., Newton R.U., Saez de villarreal E. (2014). Effects of 18-week in-season heavy-resistance and power training on throwing velocity, strength, jumping, and maximal sprint swim performance of elite male water polo players. J. Strength Cond. Res..

[13] De Villarreal E.S., Suarez Arrones L., Requena B., Haff G.G., Ramos-Veliz R. (2014). Effects of dry-land vs in-water specific strength training on professional male water polo players performance. J. Strength Cond. Res..

[14] Gorostiaga E.M., Granados C., Ibanez J., Izquierdo M. (2005). Differences in physical fitness and throwing velocity among elite and amateur male handball players. Int. J. Sports Med..

[15] Granados C., Izquierdo M., Ibanez J., Ruesta M., Gorostiaga E.M. (2013). Are there any differences in physical fitness and throwing velocity between national and international elite female handball players?. J. Strength Cond. Res..

[16] Fernandes R.J., Sousa M., Machado L., Vilas-Boas J.P. (2011). Step length and indivudual anaerobic threshold assesment in swimming. Int. J. Sports Med..

[17] Smith H.K. (1998). Applied physiology of water polo. Sports Med..

